# Accumulation of Human-Adapting Mutations during Circulation of A(H1N1)pdm09 Influenza Virus in Humans in the United Kingdom

**DOI:** 10.1128/JVI.01636-14

**Published:** 2014-11

**Authors:** Ruth A. Elderfield, Simon J. Watson, Alexandra Godlee, Walt E. Adamson, Catherine I. Thompson, Jake Dunning, Mirian Fernandez-Alonso, Deena Blumenkrantz, Tracy Hussell, The MOSAIC Investigators, Maria Zambon, Peter Openshaw, Paul Kellam, Wendy S. Barclay

**Affiliations:** a Section of Virology, Faculty of Medicine, Imperial College London, London, United Kingdom; b Section of Leucocyte Biology, Faculty of Medicine, Imperial College London, London, United Kingdom; c Section of Respiratory Medicine, Faculty of Medicine, Imperial College London, London, United Kingdom; d Virus Reference Department, Public Health England, London, United Kingdom; e Wellcome Trust Sanger Institute, Hinxton, United Kingdom; f West of Scotland Specialist Virology Centre, Gartnavel General Hospital, Glasgow, United Kingdom

## Abstract

The influenza pandemic that emerged in 2009 provided an unprecedented opportunity to study adaptation of a virus recently acquired from an animal source during human transmission. In the United Kingdom, the novel virus spread in three temporally distinct waves between 2009 and 2011. Phylogenetic analysis of complete viral genomes showed that mutations accumulated over time. Second- and third-wave viruses replicated more rapidly in human airway epithelial (HAE) cells than did the first-wave virus. In infected mice, weight loss varied between viral isolates from the same wave but showed no distinct pattern with wave and did not correlate with viral load in the mouse lungs or severity of disease in the human donor. However, second- and third-wave viruses induced less alpha interferon in the infected mouse lungs. NS1 protein, an interferon antagonist, had accumulated several mutations in second- and third-wave viruses. Recombinant viruses with the third-wave NS gene induced less interferon in human cells, but this alone did not account for increased virus fitness in HAE cells. Mutations in HA and NA genes in third-wave viruses caused increased binding to α-2,6-sialic acid and enhanced infectivity in human mucus. A recombinant virus with these two segments replicated more efficiently in HAE cells. A mutation in PA (N321K) enhanced polymerase activity of third-wave viruses and also provided a replicative advantage in HAE cells. Therefore, multiple mutations allowed incremental changes in viral fitness, which together may have contributed to the apparent increase in severity of A(H1N1)pdm09 influenza virus during successive waves.

**IMPORTANCE** Although most people infected with the 2009 pandemic influenza virus had mild or unapparent symptoms, some suffered severe and devastating disease. The reasons for this variability were unknown, but the numbers of severe cases increased during successive waves of human infection in the United Kingdom. To determine the causes of this variation, we studied genetic changes in virus isolates from individual hospitalized patients. There were no consistent differences between these viruses and those circulating in the community, but we found multiple evolutionary changes that in combination over time increased the virus's ability to infect human cells. These adaptations may explain the remarkable ability of A(H1N1)pdm09 virus to continue to circulate despite widespread immunity and the apparent increase in severity of influenza over successive waves of infection.

## INTRODUCTION

In 2009, a novel H1N1 influenza virus [A(H1N1)pdm09] crossed the species barrier from swine into humans, causing the first influenza pandemic of the 21st century. The swine-origin virus displayed a complex genotype, including antigen gene segments derived from swine-adapted influenza viruses that had previously circulated on different continents and an internal gene cassette known as the triple-reassortant genotype (TRIG), first described in pigs in the late 1990s (1–3). The TRIG cassette contained two polymerase components, PB2 and PA, from an avian virus and the other, PB1, from a human-adapted virus. The NP, HA, and NS gene segments of the pandemic H1N1 2009 virus were acquired from the classical swine virus lineage that has circulated in pigs since 1918 and had been maintained in North American swine viruses (4, 5). Classical swine influenza viruses shared an origin with the human H1N1 seasonal influenza viruses, but the two had since undergone species-specific mutations in their respective hosts. The genetic distance between the HA genes was sufficient to cause a pandemic, despite the circulation of seasonal H1 viruses in humans from 1977 until 2009. The A(H1N1)pdm09 NP gene had adaptations associated with evasion of MxA from swine or humans (6). Finally, RNA segment 8, encoding NS1 and NEP proteins, had accumulated many mutations that differentiated it from the NS segment of human-adapted influenza viruses. Notably, the swine virus NS1 protein had become truncated through a termination codon at amino acid 220 in comparison to the human-adapted NS1 protein, which retained a typical NS1 length of 230 residues (5). A functional difference in the swine-origin NS1 was reported by Hale et al. (7) and confirmed by us (8), whereby the ability to bind to the human host cell factor CPSF 30 and limit host gene expression had been lost by the accumulation of at least 3 mutations in the C-terminal domain of the NS1 gene. Thus, the virus that crossed from pigs to humans and sparked the 2009 pandemic was not optimized for human replication and transmission because its gene segments were swine adapted.

In the United Kingdom, there were two waves of A(H1N1)pdm09 activity during the 2009-2010 pandemic period: an initial out-of-season outbreak that started in April 2009 and peaked in July 2009, followed by a second wave in the autumn and winter of 2009-2010. In the first postpandemic winter (2010-2011), a third wave of A(H1N1)pdm09 activity was seen. This third wave was associated with an increase in infection and severity and a shift in age demographics from children (0 to 15 years old) and younger adults (16 to 44 years old) to predominantly adults (9–12). Compared with the first two pandemic waves, the third wave was associated with more hospital admissions (8,797 versus 7,879 people), more people admitted to critical care (2,200 versus 1,700 people), and a greater number of deaths (474 versus 361 people) in England (13). Although there had been evidence of sequence variation in viruses in the second pandemic wave in the United Kingdom and elsewhere (14, 15), surveillance and antigenicity studies had reported no change in the antigenicity of the surface glycoproteins hemagglutinin (HA) and neuraminidase (NA), so a vaccine update was not warranted (14). Moreover, any change in antigenicity would be unlikely to explain the increased severity of third-wave viruses in the unvaccinated or those who had not previously contracted the virus. Nonetheless, it is possible that other genetic changes distinct from those with an antigenic effect may have led to increased circulation of the virus or enhanced virulence that accounted for the apparent increased severity in the United Kingdom's third wave. Indeed, Dorigatti and Ferguson recently modeled the United Kingdom third wave and concluded that the observed increased numbers of cases were most likely accounted for by an increase in virus transmission with a commensurate increase in the numbers of severely ill. They suggested this could be due to weather conditions, as that winter was particularly cold and dry and favored virus transmission events, or to a change in the inherent transmissibility of the virus itself, or both (16). Here we report genetic variability across the three waves of influenza virus A(H1N1)pdm09 in the United Kingdom and identify nonsynonymous variants that define the third-wave viruses. We show that mutations in HA and NA, the PA component of the polymerase complex, and the NS1 interferon (IFN) antagonist protein enhanced the virus' ability to replicate in human airway cells.

## MATERIALS AND METHODS

### Cells.

Madin-Darby canine kidney (MDCK), human embryonic kidney (293T), and newborn pig tracheal (NPTr) cells were grown in Dulbecco's modified Eagle medium (DMEM) supplemented with 10% fetal calf serum (FCS). MucilAir cultures of human nasal epithelium (HAE; Epithelix) were grown in MucilAir medium. All cells were maintained at 37°C in 5% CO_2_.

### Patient recruitment and sampling.

The MOSAIC study investigators recruited adult and pediatric patients admitted to the hospital with suspected influenza virus infection in London and Liverpool between December 2009 and February 2011. Infection with seasonal influenza A H3N2, influenza B, or A(H1N1)pdm09 viruses was confirmed locally by viral PCR according to regional protocols. Patients were approached for recruitment and initial (T1) sampling as soon as possible following admission to a MOSAIC-associated hospital. Nasopharyngeal aspirate (NPA) and viral throat swab samples were obtained according to the study standard operating procedures. Patients with comorbidities were not excluded. Different severities of illness were included, and severity was graded as follows: grade 1, no respiratory compromise (oxygen saturation of >93% on room air); grade 2, respiratory compromise requiring noninvasive oxygen supplementation; grade 3, respiratory compromise requiring invasive mechanical ventilation and oxygen supplementation. The MOSAIC study was approved by the NHS National Research Ethics Service, Outer West London REC (09/H0709/52 and 09/MRE00/67).

### Viruses.

Influenza viruses were isolated from clinical specimens by the Respiratory Virus Unit, Public Health England (PHE), Colindale, London. Briefly, clinical specimens, including NPA, viral throat swab in virus transport medium (VTM), bronchial alveolar lavage (BAL) fluid, or endotracheal aspirate (ETA), were transported to PHE frozen on dry ice. For virus isolation, 200-μl clinical specimen aliquots were inoculated onto monolayers of MDCK cells or the SIAT-1 cell derivative in virus isolation tubes and allowed to adsorb for 1 h (17). Cells were incubated in serum-free Earles minimal essential medium in the presence of 1.25 μg/ml tosylsulfonyl phenylalanyl chloromethyl keton (TPCK)-treated trypsin (Worthington) on a rolling drum at 33°C for a maximum of 7 days with regular observation for viral cytopathic effect (CPE). Blind passage on fresh cells for a further 7 days was performed where necessary. Virus growth was determined by hemagglutination assay using turkey or guinea pig red blood cells. One further virus passage was made to generate a large stock of virus for distribution to the MOSAIC group.

### Plasmid-based reverse genetics.

The reverse genetics viruses were generated as previously described (8) from plasmids either synthesized (GeneArt) from the A/England/195/2009 whole-genome sequence and A/England/687/2010 segment 4 sequence or generated by site-directed mutagenesis (Stratagene Lightening mutagenesis kit) of A/England/195/2009 plasmid sequence with the point mutations necessary to create the A/England/687/2010 amino acid sequence. The plasmids were sequenced to confirm the presence of the required mutations and absence of unwanted variations. Primer sequences are available upon request. Reverse genetics viruses were generated using the 12-plasmid system with either A/England/195/2009 or A/England/687/2010 polymerase I clones and helper polymerase of A/Victoria/3/75.

### Virus replication in cell lines and primary airway cultures.

Confluent cell monolayers were infected with equal PFU of each virus at a multiplicity of infection (MOI) of 0.01 or 0.001 as indicated below. The cells were incubated in the inoculum for 1 h, and then the inoculum was removed, cells were washed with phosphate-buffered saline (PBS), overlaid with DMEM supplemented with nonessential amino acids, penicillin-streptomycin, and TPCK-treated trypsin, and incubated at 34°C in 5% CO_2_. Growth was assessed by well sampling at fixed time points and titration on MDCK cells by plaque assay. For infection of MucilAir cultures, the apical surface (air interface) was washed with serum-free (SF) medium prior to infection, then washed again after the inoculation. Viral titer was assessed by sampling from the apical surface by the addition of 200 μl of serum-free medium, incubation for 15 min, and removal of the medium. The basal layer was sampled for cytokine analysis, with an equal volume of MucilAir medium replacing that removed.

### Virus competition assays.

Cells in MucilAir were washed with serum-free DMEM prior to inoculation with a 50:50 mix of two viruses at a total MOI of 0.001. The cells were incubated for 1 h prior to removal of the inoculum and washing with SF medium. The apical layer was sampled every 12 h as described above. Viral RNA was extracted from the supernatant with a Qiagen Qiamp vRNA kit and processed for Ilumina deep sequencing. Each assay was run in triplicate.

### Minigenome polymerase assays.

The coding sequences for the PB1, PB2, PA, and NP proteins were amplified using KOD polymerase (Novagen) and primers containing restriction sites to allow incorporation into pCAGGs expression vectors. To introduce alternative amino acids into the coding sequence, site-directed mutagenesis was undertaken on the reverse genetic genomic plasmids. Primers are available on request. pCAGGs expression plasmids for PB1, PB2, PA, and NP proteins were transfected onto a confluent layer of 293T cells by using Lipofectamine 2000 and Optimem in a plasmid concentration ratio of 1:1:0.5:2, respectively. Additionally, a plasmid encoding a minigenome firefly luciferase reporter flanked by the promoter region of the influenza virus segment 8 and either a Renilla or β-galactosidase transfection control plasmid were cotransfected with the minigenome complement. Cells were incubated with the transfection mix for 24 h at 34°C at 5% CO_2_. Then, supernatant was removed, the cells were washed, and 100 μl of passive lysis buffer was added (Promega). The cells were freeze-thawed, and lysates were analyzed with the dual luciferase reporter system (Promega) on the FLUOstar Omega system (BMG Labtec). All assays were run in triplicate, with each assay being repeated a minimum of 3 times.

### Interferon reporter assays.

A plasmid with the beta interferon promoter region upstream of a firefly luciferase reporter was transfected along with a β-galactosidase or Renilla control plasmid into 293T cells in suspension by using Lipofectamine 2000 and Optimem. 293T cells were allowed to adhere to plates pretreated with poly-l-lysine and incubated overnight. The cells were then infected with virus at an MOI of 3 and incubated at 37°C for 1 h prior to inoculum removal, washing, and overlaying with DMEM with 10% FCS. The cells were then harvested at set time points by the removal of medium, washing with PBS, the addition of passive lysis buffer, and passage through a freeze-thaw cycle prior to detection with the dual luciferase reporter system (Promega) on the FLUOstar Omega apparatus (BMG Labtec). All assays were run in triplicate, with each assay being repeated a minimum of 3 times.

### Mouse experiments.

All animal procedures and care conformed strictly to the United Kingdom Home Office Guidelines under the Animals (Scientific Procedures) Act 1986, and the protocols were approved by the Home Office of Great Britain (license number 70/6646).

Fifteen weight-matched female BALB/c mice anesthetized with isoflurane were inoculated with 7.5 × 10^5^ or 2 × 10^5^ PFU of each influenza virus. Mice were weighed daily, and any mice falling below a 30% weight loss threshold were sacrificed. At day 2 and day 4 postinfection, 5 mice from each experimental group were sacrificed and the lungs harvested. Whole lungs were weighed and homogenized in the presence of 1 ml of PBS and split into aliquots. An aliquot of lung homogenate from each mouse was subjected to titration by plaque assay. Results are expressed in milliliters of the homogenate, as whole mouse lungs were homogenized in a standardized volume of PBS.

### Interferon detection.

Aliquots of mouse lung homogenate were tested for the presence of mouse interferon by using the Verikine alpha interferon kit (R&D Systems) according to the manufacturer's instructions and then measured on the FLUOstar Omega system (BMG Labtec). Each sample was run in duplicate.

### Phylogenetic analysis.

Complete genomes of samples sequenced by the MOSAIC group were aligned against all complete A/H1N1/09 genomes up to 2011 that were present in the NCBI Influenza Virus Resource database (18). To improve tree readability, this set of 2,084 genomes was down-sampled by using custom Python scripts, ensuring that the topology of the phylogenetic tree was maintained. Phylogenetic trees were inferred by using a neighbor-joining clustering method with branch lengths and substitution parameters estimated using the Tamura-Nei model under the maximum composite likelihood method implemented in MEGA version 6.06 (19). Tree robustness was evaluated by bootstrapping with 1,000 pseudoreplicates.

### Statistical analysis.

All statistical analyses were conducted using GraphPad Prism software. Virus replication, IP-10, IFN cytokine production, and mucus inhibition assays were assessed by unpaired *t* tests compared to the A/195 first-wave isolate. The interferon and minigenome assays were assessed by repeated-measures or ordinary one-way analysis of variance (ANOVA) with Tukey's multiple-comparison test, as appropriate.

## RESULTS

The Mechanisms of Severe Acute Influenza Consortium (MOSAIC) was formed to investigate why some individuals infected by the pandemic H1N1 influenza virus developed severe symptoms requiring hospitalization while others developed a milder coryzal illness. MOSAIC recruited 85 patients admitted to the hospital with influenza-like illness in the winter of 2009-2010 and a further 172 patients in the winter of 2010-2011. NPA and viral throat swab specimens were collected from patients for attempted isolation of virus strains by culture in MDCK cells, assessment of viral titer by quantitative reverse transcription-PCR (qRT-PCR), and whole-genome sequencing directly from the clinical sample.

### Evolution of influenza A(H1N1)pdm09 viruses showed a distinct United Kingdom third-wave lineage.

**FIG 1:**
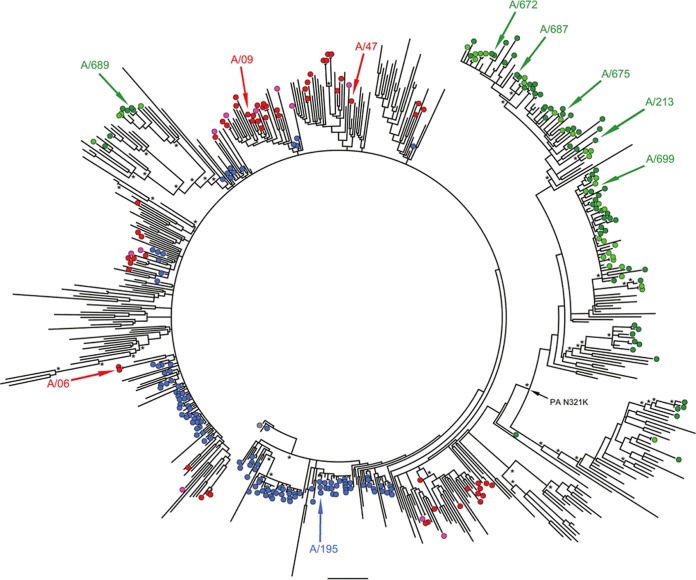
Phylogenetic relationship of complete influenza A(H1N1)pdm09 virus genomes. The tree is rooted on A/California/04/2009, shown as a blue-filled circle. United Kingdom first-wave isolates are highlighted as blue circles, while isolates sequenced by MOSAIC are shown as red circles for second-wave isolates from community and hospitalized patients, and green circles show isolates from third-wave community and hospitalized patients. Isolates characterized in this study are indicated with colored arrows, while the inferred ancestral location of the asparagine-lysine mutation in PA is indicated with a black arrow. Nodes with bootstrap support of >75% are highlighted with asterisks. Bar, 0.002 substitutions/site.

Clinical samples, either NPA or throat swabs that were positive for influenza A(H1N1)pdm09 RNA, were prepared without virus isolation or passage and sequenced by using either the Roche Genome Sequence FLX 454 or Illumina Genome Analyzer IIx platforms as previously described (20) and assembled into full genomes as described elsewhere (21). Background whole-genome pandemic sequences were downloaded from NCBI's Influenza Virus Resource database (18) and aligned against the United Kingdom first-, second-, and third-wave genomes. Phylogenetic analysis showed that viruses from the first two pandemic waves were closely related, consistent with the proposed persistence of the first-wave lineages into the second wave in the United Kingdom (20). However, viruses detected during the third wave (winter of 2010-2011) were genetically distinct, with the majority of genomes clustering in a separate monophyletic clade (Fig. 1). Across the second and third waves in the United Kingdom, viruses from hospitalized patients were phylogenetically indistinguishable from community and nonhospitalized control patients and did not contain shared genome variants that could confer enhanced pathogenicity (Fig. 1). We assessed amino acid changes fixed in the majority of viruses and identified 21 common changes across all segments, of which 12 were unique to third-wave viruses (Table 1). These changes also accumulated in second- and third-wave virus isolates from other regions of the United Kingdom and from the rest of the world (Table 2), except for G189D in NS1. We also checked for nucleotide differences in segment 2 that would affect the translation of PB1-F2 and/or N40 open reading frames, but we found none.

**TABLE 1:**
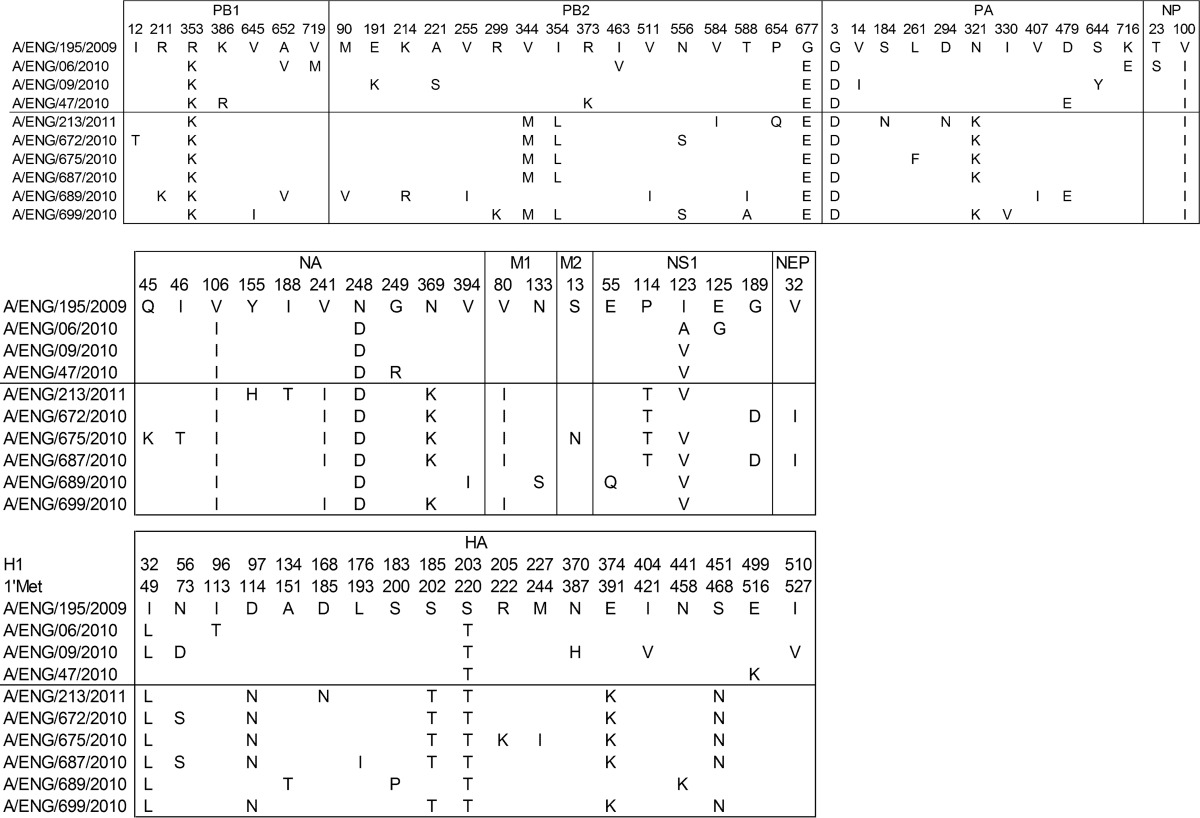
Amino acid changes observed in 10 A/H1N1 pdm(2009) viruses isolated in the United Kingdom, in comparison to prototypic virus A/England/195/2009^*a*^

a Data for the three isolates from the 2009-2010 season are above the line, and data for the 6 isolates from 2010-2011 are below it.

**TABLE 2:**
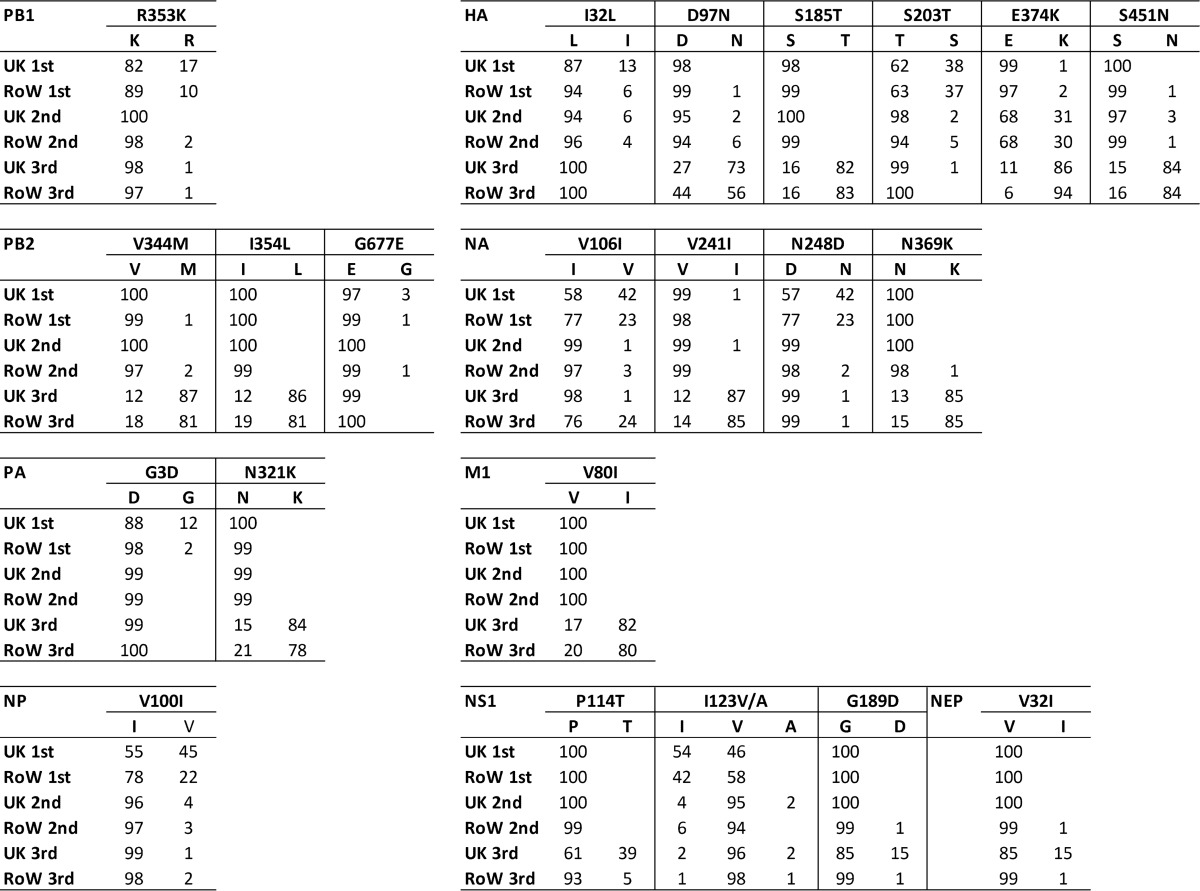
Prevalence ratios of amino acid changes in influenza viruses in the United Kingdom and the rest of the world (RoW) during the first, second, and third waves of virus infection

a Incomplete ratios are due to unknown or multiple minor populations. HA numbering is based on that of H1.

### Clinical isolates from the first, second, and third pandemic waves varied in their growth kinetics in primary human airway cultures.

The lack of a common genetic difference between influenza viruses isolated from severe or mild influenza cases in each wave suggests that the observed increase in overall severity in the third wave may be related to a general property of third-wave viruses, which should be reflected in virological differences between first-, second-, and third-wave viruses.

To assess virus differences, we isolated virus from clinical samples in SIAT-MDCK cells for a subset of viruses selected in accordance with their placements on the phylogenetic tree in Fig. 1. We infected MDCK cells or primary HAE cultures or mice with 9 different clinical isolates from the second wave (3 viruses) and the third wave (6 viruses), and we compared growth and outcome of infection with the first-wave prototypic United Kingdom A(H1N1)pdm09 virus, A/England/195/2009. Clinical data for patients from whom these isolates were obtained are shown in Table 3. We recorded the differences in viral RNA load in NPA at time of recruitment (T1 NPA titer), the severity score for the patient, and the presence of patient comorbidities. With the caveat that the delay between symptom onset and recruitment varied from patient to patient (day since symptom onset), we did not find any correlation between viral load and severity score for this subset of the MOSAIC cohort.

**TABLE 3:** Patient information for the MOSAIC viral isolates

Isolate	Severity score^*a*^	Comorbidities^*b*^	Collection date (mo-yr)^*c*^	Days since symptoms onset^*d*^	Virus detection^*e*^	T1 NPA titer^*f*^ (PFU/ml)
A/England/195/2009	Mild	NK^*g*^	Apr-09	NK	NK	NK
A/England/06/2010	1	Yes	Jan-10	20	NPA	5,802
A/England/09/2010	1	Yes	Dec-09	2	NPA	7,035
A/England/47/2010	3	Yes	Dec-09	9	NPA, blood, stool	2,315
A/England/213/2011	2	Yes	Jan-11	1	NPA	47
A/England/672/2010	2	Yes	Dec-10	4	NPA	0.2
A/England/675/2010	1	No	Dec-10	2	NPA	2
A/England/687/2010	2	Yes	Dec-10	2	NPA	8
A/England/689/2010	1	Yes	Dec-10	2	NPA	1,251
A/England/699/2010	3	Yes	Dec-10	4	NPA	23

a The severity score was assigned according to the severity of respiratory impairment, as follows: grade 1, no respiratory compromise (oxygen saturation of >93% on room air); grade 2, respiratory compromise requiring noninvasive oxygen supplementation; grade 3, respiratory compromise requiring invasive mechanical ventilation and oxygen supplementation.

b Predisposing (comorbid) conditions included asthma, immunosuppression, etc. NK, not known.

c The month the sample (from which the virus was isolated) was collected.

d The time between onset of symptoms and viral sample collection.

e Sample types in which virus was detected. NPA, nasopharyngeal aspirate.

f The viral titer was assessed by qPCR of the H1N1 NA gene and compared against a standard curve of viral RNA of known PFU/ml.

g NK, not known.

**FIG 2:**
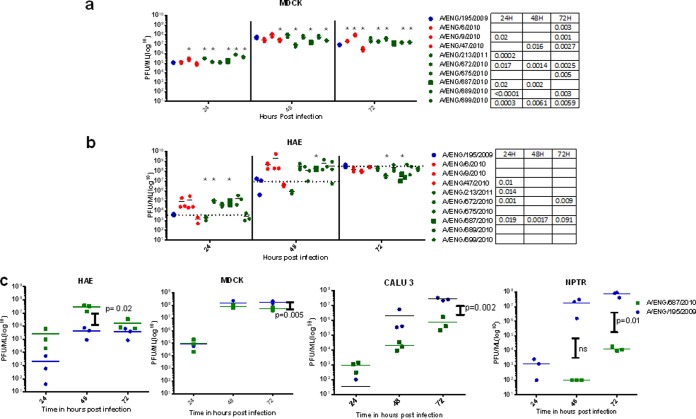
Replication of A(H1N1)pdm09 viruses in cell culture. (a and b) Viral growth of 10 clinical isolates from first-wave (blue), second-wave (red), or third-wave (green) viruses in MDCK cells (a) and human nasal MucilAir cell cultures (HAE; Epithelix) (b). Cells were infected at an MOI of 0.001 (MDCK cells) or 0.01 (MucilAir) and incubated at 34°C. The dashed line represents the mean for A/195. Statistics in the tables adjoining the chart keys were calculated using unpaired *t* tests with Holm-Sidak corrections. (c) Replication of a representative first-wave (A/195; blue circles) and third-wave (A/687; green squares) virus pair was assessed in HAE cells (left panel), MDCK cells (left middle panel), CALU3 cells (right middle panel), and pig tracheal cells (right panel). Cells were infected at an MOI of 0.001. Statistics were calculated using unpaired *t* tests.

*In vitro*, first-, second-, and third-wave viruses used to infect MDCK cells did not show a consistent difference in replication pattern according to respective waves, although there were small variations between individual isolates (Fig. 2A). In HAE cell cultures, the patterns of virus replication were more diverse and correlated with respective waves. Although two viruses, one from the second wave (A/47) and one from the third wave (A/213), displayed similar growth kinetics to the prototypic A/195 virus, most second- and third-wave viruses displayed a growth advantage at 24 and 48 h postinfection in human airway cells. Using a different HAE culture code (primary cells obtained from a different donor), a representative third-wave clinical isolate, A/687, displayed a consistent 2-log_10_ growth advantage at the 24- and 48-h time points, with a peak titer of 10^8^ PFU/ml, compared with 10^6^ PFU/ml for the A/195 first-wave virus at 48 h postinfection (*P* = 0.02), before the virus titers became similar at 72 h postinfection (Fig. 2c). Strikingly, in pig tracheal cell cultures (NPTr), the third-wave virus A/687 was compromised in growth (Fig. 2c). In other human lung cell lines, such as Calu-3, the first-wave virus also replicated more efficiently than the third-wave virus, although the difference was not as pronounced as in the pig cell line. This suggests that the adaptation in third-wave virus might be conferred by features only present in the well-differentiated complex HAE cultures.

### Outcomes of infection in the *in vivo* BALB/c mouse model did not correlate with patient severity or growth in human airway cultures.

**FIG 3:**
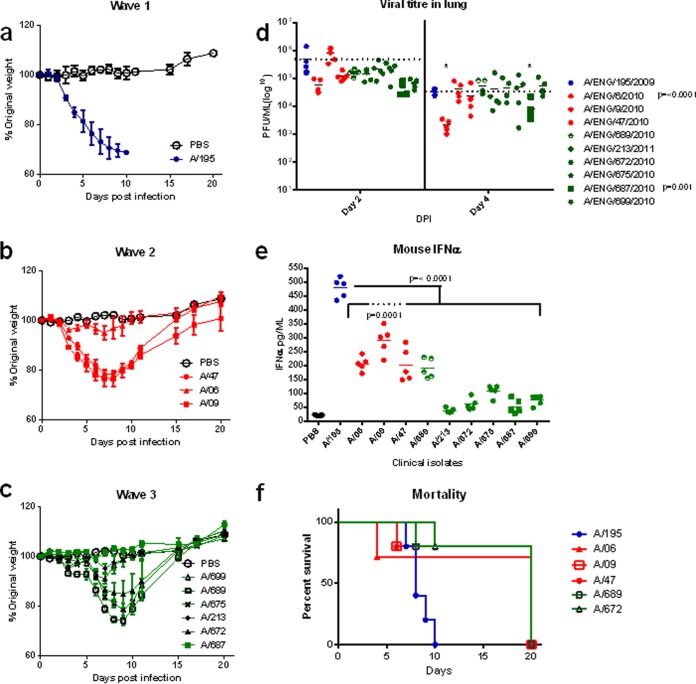
Infection of mice with A(H1N1)pdm09 viruses. (a to c) Weight loss was followed after infection of 15 BALB/c mice inoculated intranasally with 2 × 10^5^ PFU of virus isolates: (a) one A/195 first-wave isolate (blue); (b) three second-wave isolates (red); (c) six third-wave isolates (green). Weights in mice inoculated with PBS are shown in black. (d and e) Virus titers (d) and interferon levels (e) in lung homogenates at day 2. (f) Mouse mortality data for virus infections where weight loss necessitated culling.

In order to assess the *in vivo* characteristics of this panel of clinical isolates, we utilized a mouse model. BALB/c mice were infected intranasally with each of the panel of viruses. After a dose of 2 × 10^5^ PFU, the A/195 first-wave isolate caused >20% weight loss by day 5 postinfection (Fig. 3a). Some of the second- and third-wave viruses inoculated at the same dose were less pathogenic in mice, as ascertained by less weight loss; for example, the A/687 third-wave virus induced only 2% weight loss and no mortality (Fig. 3b and c). Mouse mortality (due to humane cull based on weight loss) was observed in all three of the sets of mice infected with the second-wave viruses but only in two sets (A/689 and A/672) of the third-wave virus-infected mice. Statistically, only the A/09-infected mice did not show a difference in weight loss compared to the A/195-infected mice; the A/47 and the A/689 mouse groups displayed a statistical difference only on days 2 and 3, respectively. The differences in weight loss and mortality in mice did not correlate with the severity scores assigned to the human patients infected with the same virus (Fig. 3a, b, and c and Table 3). Interestingly, the viral loads in the mouse lungs at day 2 or 4 postinfection did not show the same pattern as titers in HAE culture infections, with a trend toward lower virus lung titers at day 2 for the second- and third-wave viruses compared to A/195 (Fig. 3d). For example, the first-wave A/195 virus replicated to high levels in mice, whereas the third-wave A/687 virus replicated comparatively poorly, but in HAE cells the situation was reversed (Fig. 2c). However, in some cases the reciprocal pattern between HAE replication and mouse pathogenicity was not maintained: A/672, which had similar growth in HAE as A/687, replicated to a higher titer, induced greater weight loss of around 15%, and led to 20% mouse mortality. There are only four coding genetic differences between these two third-wave viruses: HA L176I, NS1 I123V, PB1 I12T, and PB2 N556S.

The most obvious trait that was associated with virus wave was a clear association between the IFN-α level in the mouse lung and the wave of isolation for each virus; levels of IFN in lungs of mice infected with second-wave viruses were lower than for A/195, and the lowest IFN levels were in third-wave virus-infected mouse lungs (Fig. 3e). The lower IFN production as the waves progressed could reflect the virus adapting to better control the host immune system. The same patterns of weight loss, lung titers, and lung interferon levels for each isolate were observed in a separate experiment in which mice were infected with 7.5 × 10^5^ PFU (data not shown).

### Virus with segment 8 of the third-wave virus induced less type I interferon.

**FIG 4:**
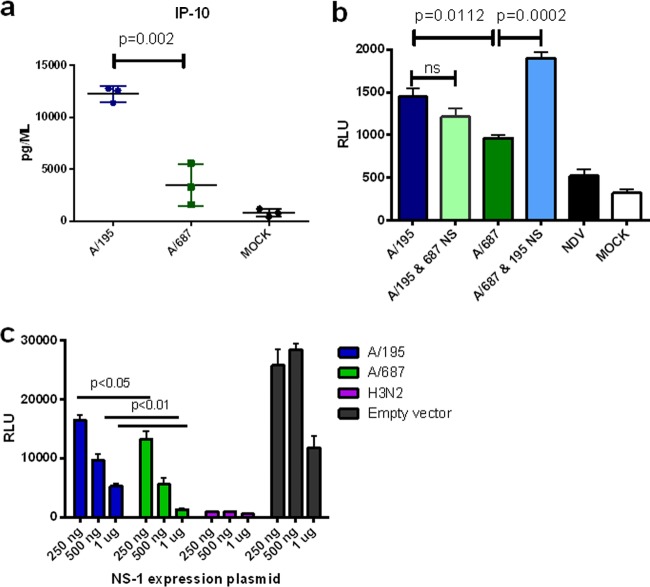
Cytokine induction by first- and third-wave viruses and the role of NS gene segments in virus replication in HAE cells. (a) Human nasal MucilAir cell cultures (HAE; Epithelix) were infected in triplicate with RG A/195 and A/687 virus at an MOI of 1 for 16 h. The basal medium was harvested, and levels of the cytokine IP-10 were measured using mesoscale discovery (MSD) plates. Statistical analysis was performed with an unpaired *t* test. (b) 293T cells transiently transfected with a beta interferon promoter luciferase reporter plasmid were infected with RG viruses A/195 (dark blue), A/195 with A/687 segment 8 (light green), A/687 (dark green), and A/687 with the A/195 segment 8 (light blue) at an MOI of 3. Infection with NDV was used as a positive control. Statistical analysis entailed a one-way ANOVA, with Tukey's multiple-comparison test (and associated adjusted *P* values). (c) 293T cells transiently transfected with an beta interferon promoter luciferase reporter and pCAGGs NS1 plasmids A/195 (blue), A/687 (green), and H3N2 (purple). Positive controls were stimulated with NDV. Statistical analysis was performed with a one-way ANOVA and Tukey's multiple-comparison test.

The increased interferon in lungs of mice infected with first-wave virus might be driven by higher viral loads. However, viral loads in lungs at day 2 were not always higher in mice infected by second-wave versus third-wave viruses, but IFN levels were higher (Fig. 3d and e). We hypothesized that mutations in the virus between the second and third waves may have enhanced the virus' ability to control the innate immune response. NS1 protein encoded on RNA segment 8 is the major interferon antagonist of influenza A virus. Sequence analysis showed third-wave viruses possessed a cluster of amino acid changes in the NS1 protein in various combinations at positions E55Q, P114T, I123V, and G189D (Table 1). A third-wave isolate, A/687, possessed three of these changes within the effector domain of the NS1 protein: 114T, 123V, and 189D. The G189D variation also changed the coding sequence of the NEP protein (V32I), because the NS1 and NEP open reading frames overlap at this region. Under the conditions used for these assays, IFN-β was undetectable in apical washes or in basal medium from infected HAE cultures. Therefore, we assessed the virus' ability to counteract the production of an innate induced cytokine, IP-10. At 16 h postinfection with a high multiplicity of virus, significantly less IP-10 was secreted from HAE cells infected with the A/687 virus than cells infected with the first-wave virus A/195 (Fig. 4a). Levels of two other cytokines, interleukin-6 (IL-6) and IL-8, were also lower in basolateral media following infection of the HAE cells with third-wave virus (data not shown), but the difference did not reach significance. IFN was not detectable in samples collected from the HAE cells after infection with either virus.

To further assess the role of the NS gene mutations in controlling the cytokine response, we generated recombinant viruses with the segment 8 RNAs exchanged. The A/195 reverse genetics (RG) system described previously was generated by synthesizing the cDNAs for this strain *de novo* (8, 22). The A/687 reverse genetics virus was created by site-directed mutagenesis of the A/195 plasmids wherever an amino acid change was present, for seven of the gene segments. The gene segment encoding the A/687 HA was synthesized *de novo*.

293T cells transiently transfected with an IFN-β promoter luciferase reporter construct were infected with each of the 7:1 single-gene reassortant viruses or with the isogenic A/195 or A/687 wild-type RG viruses (Fig. 4b) (23). The A/687 RG virus induced a significantly lower luciferase signal than A/195. The induction of the IFN-β promoter was decreased relative to that with the isogenic A/195 virus when the 687 NS gene was present and significantly increased for the A/687 virus with A/195 NS.

Since there was also a difference in NEP coding between these two viruses, we tested whether the NS1 protein itself had altered activity as an interferon antagonist when expressed exogenously. At 3 different doses of NS1 expression, the third-wave NS1 protein was significantly better able to control the expression of luciferase driven by an interferon promoter in Newcastle disease virus (NDV)-infected cells (Fig. 4c).

### The HA and NA genes of third-wave viruses displayed different receptor binding preferences, enhanced infectivity in human mucus, and conferred enhanced growth in HAE cell cultures compared to first-wave viruses.

**FIG 5:**
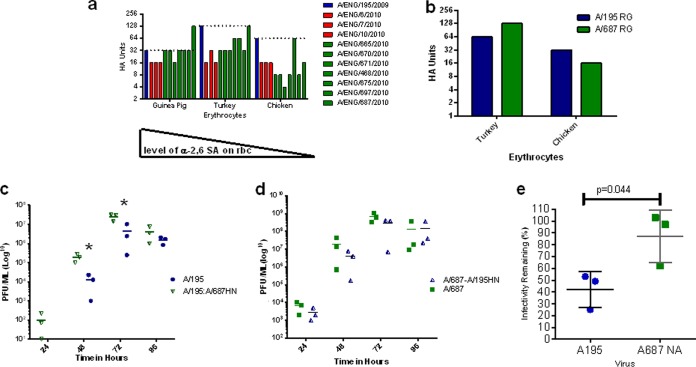
Variation in surface genes HA and NA in third-wave virus isolates leads to altered receptor binding, enhanced replication in HAE cells, and enhanced infectivity in mucus. (a) Hemagglutination assay with 11 clinical isolates from first-wave (blue), second-wave (red), and third-wave (green) isolates, assessed for binding to 0.5% chicken, turkey, or guinea pig red blood cells. The dashed line represents the A195 HA score. (b) Hemagglutination assay with equal PFU of A/195 first-wave (blue) and A/687 third-wave (green) RG viruses with 0.5% chicken or turkey red blood cells. (c) Viral replication in human nasal MucilAir cell cultures (HAE; Epithelix) of RG viruses based on A/195 with HA and NA from A/195 first-wave (blue) or HA and NA of A/687 third-wave (green triangle) isolates. (d) A/687 (green square) or A/687 with A/687 with HA and NA from A/195 (blue triangle). Cells were infected at an MOI of 0.01. *, *P* < 0.05 based on an unpaired *t* test. (e) Mucus inhibition assay. An equal PFU of A/195 (blue) or A/195 with A/687 third-wave isolate NA (green) RG virus was incubated with diluted human mucus for 1 h prior to infection of MDCK cells. Infectivity remaining was plotted as the percentage of the titer in the absence of mucus. *, *P* = 0.044 by unpaired *t* test.

Second- and third-wave United Kingdom viruses displayed wave-associated amino acid changes in the HA and NA proteins (Table 1). A collection of 11 MOSAIC virus isolates representative of the three waves, including A/195 (first wave), A/06 (second wave), and A/675 and A/687 (third wave) from the panel of nine viruses studied above, were tested in a hemagglutination assay using erythrocytes from different species (guinea pig, turkey, and chicken), which are known to differ in specificity and density of sialic acids expressed on their cell surfaces (24). All viruses displayed comparable binding to guinea pig and turkey erythrocytes, but the second- and third-wave isolates displayed lower binding to chicken red blood cells relative to A/195 (Fig. 5a). The only exception was A/675, which has an M227I (230 in H3 numbering) amino acid variation near the receptor binding pocket and retained strong binding to chicken erythrocytes. HA assays performed with RG A/195 and A/687 viruses recapitulated the general pattern seen for the panel of isolates; the relative binding of the first-wave virus for chicken erythrocytes was higher than for the third-wave virus (Fig. 5b).

A/687 virus differs from A/195 by 8 amino acids in the HA protein and 4 in the NA protein. To test if the changes in HA and NA alone were sufficient to confer the observed growth advantage in human airway cells (Fig. 2b and c), we generated 6:2 recombinant viruses with the A/687 HA and NA combined with A/195 internal proteins or the A/687 internal genes with the HA and NA of A/195, and we compared their replication with that of isogenic wild-type RG A/195 or RG A/687. The virus with the A/687 internal genes coupled with first-wave HA and NA replicated to a lower titer than whole A/687 virus, and conversely, the virus with A195 internal genes and third-wave HA and NA replicated to higher titers than whole A/195 virus. However, the differences in replication did not reach statistical significance (Fig. 5c). This suggests that both internal and external genes contribute to the enhanced replication of third-wave virus in HAE cultures, but neither of the gene sets is sufficient to reproduce the phenotype alone.

To investigate a role for the third-wave NA protein in enhanced replication in HAE cultures, we generated a 7:1 RG virus with 7 segments from the A/195 virus and RNA segment 6, which encodes the NA protein from A/687. This virus and the isogenic A/195 virus were then incubated in the presence of human respiratory mucus for 60 min prior to infection of MDCK cells. The numbers of plaque-forming foci were counted, and the percent reduction in infectivity was calculated (25). The virus carrying the third-wave NA gene segment displayed an increased ability (*P* = 0.044) to overcome inhibition of infectivity by human mucus, which is an important factor in the ability of a virus to infect and spread in the human airway (Fig. 5d).

### The mutation in PA N321K of third-wave viruses conferred enhanced replication in a minireplicon assay and in viral competition assays.

Mutations in other genes of the second- and third-wave viruses may have also contributed to human adaptation and enhanced replication in HAE cells. In the polymerase proteins and nucleoprotein of the A/687 virus, there were 7 amino acid changes from A/195 polymerase genes, 3 of which were unique to the majority of the third-wave viruses in the United Kingdom. These three changes were at PB2 V344M, I354L, and PA N321K (Table 1).

**FIG 6:**
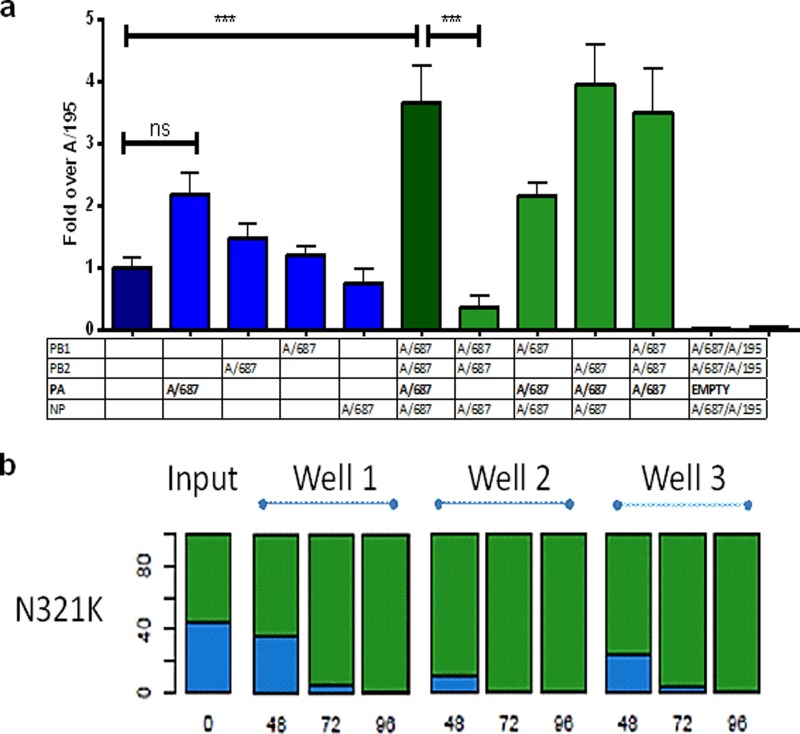
PA of third-wave virus confers enhanced polymerase activity and a fitness advantage in HAE cells. (a) Activities of polymerase reconstituted from plasmids expressing polymerase components of A/195 first-wave (dark blue) and A/687 third-wave (dark green) viruses. 293T cells were transiently transfected with a plasmid that directs *in situ* synthesis of a minigenome in which a luciferase reporter gene is flanked by the influenza A virus promoter. Cotransfection of a Renilla expression plasmid was used to normalize for transfection efficiency. Combinations of the PB1, PB2, PA, and NP expression plasmids of A/195 (blue) or A/687 (green) or lacking the PB2 polymerase (white) were transfected. At 24 h posttransfection, cells were harvested. The results were normalized based on Renilla results (transfection control) and are from three separate set of experiments, each with triplicate wells (*n* = 9). Differences were analyzed using a one-way ANOVA test with Tukey's multiple-comparison test. (b) Human nasal MucilAir cell cultures (HAE; Epithelix) were infected in triplicate with RG viruses based on A/195 that differed only in PA at N321K. Virus released was harvested at 24, 48, 72, and 96 h and sequenced using the Illumina system. The percentage of N allele (first wave) is represented in blue and K (third wave) is shown in green.

In order to assess whether these amino acid changes altered the activity of the viral polymerase, we created two sets of expression plasmids that allowed reconstitution of polymerase components from either A/195 or from A/687 virus. For the A/195 constellation, we used a PA plasmid with the glycine 3 mutated to aspartic acid, since this difference was atypical among first-wave viruses (17). Each viral polymerase was reconstituted in a minireplicon reporter assay in 293T cells as previously described (26). The third-wave A/687 polymerase and nucleoprotein complex consistently directed higher amplification and higher expression of the luciferase signal from the minireplicon in human cells than the A/195 polymerase constellation (*P* ≤ 0.001) (Fig. 6a).

In order to discern if this enhancement of polymerase activity was a result of amino acid changes in a single protein, the minireplicon assays were carried out with single protein exchanges. A dramatic 10-fold decrease in signal was observed when the A/195 PA protein was paired with the A/687 PB1, PB2, and NP proteins. The reciprocal exchange showed the converse result, although the increase in polymerase activity when A/687 PA was introduced into the A/195 polymerase constellation was not statistically significant (Fig. 6a).

To assess whether the PA N321K mutation led to an enhancement of replication in the context of whole virus, we generated a 7:1 (PA) RG virus in which every genomic segment except for the A/687 PA segment was derived from A/195. Replication competition experiments starting with a 50:50 mix in triplicate HAE cultures were analyzed by deep sequencing using Illumina sequencing technology and showed the virus with the PA 321K from the third-wave genotype rapidly dominated the viral RNA population in all 3 biological replicates (*P* = 0.0078, <0.0001, and <0.0001 at 48, 72, and 96 h, respectively, when assessed as the percentage of total reads via an unpaired *t* test), illustrating a growth advantage in human cells conferred by this amino acid change (Fig. 6b).

## DISCUSSION

In the United Kingdom, there was a reported increase in severity of the pandemic H1N1 virus as the waves of influenza virus infection progressed from its emergence in spring 2009 until the end of winter 2011 (10, 12). It has been suggested that this can be attributed to a shift in the behavior of the infected population due to a change in public health responses (10, 13). However, this explanation does not exclude host adaptations in the influenza virus itself that might also have contributed to the change in severity associated with A(H1N1)pdm09 infection. If the virus were responsible, there may have been a specific virulence factor that appeared in many or all of the hospitalized cases; however, we found no evidence for this in our study. We can discern from whole-genome sequence data provided by FLUWatch, FF100, PHE, RCGP, and the isolates derived from the hospitalized MOSAIC patients over three waves of infection in the United Kingdom that the viruses derived from hospitalized patients did not vary genetically from those found within the community, consistent with the findings of Galliano et al. for the second wave (27).

Alternatively, a constellation of adaptive changes that accumulated in the virus as it circulated in the community could have made it more likely that a higher proportion of individuals would become infected, with possibly more efficient early virus replication, leading to more severe infections in some people.

A great deal of effort worldwide has been put into tracing amino acid changes in the pandemic H1 HA, and to a lesser degree the NA (28–37). This emphasis on the glycoproteins is understandable, due to their antigenic properties and importance in determining antiviral susceptibility. Indeed, various functional consequences of naturally occurring amino acid changes in the NA and HA proteins have already been demonstrated using animal models (38–43). In our MOSAIC hospitalized cohort, we did not detect the specific HA mutations described by others to affect A(H1N1)pdm09 virulence, for example, HA D222G, which purportedly facilitates better binding to α-2,3-sialic acid-linked receptors in the lower respiratory tract (44–47), changes in glycosylation (48), or antigenicity (49) were not present. Other groups have suggested that HA D222G is relatively common in severe cases (44–47).The D222G mutation has been suggested to sometimes arise during virus culture as an artifact and may also only be present as a minority variant in samples taken at certain times during the infection. We do not know why it was not detected in our MOSAIC cohort.

We detected a different type of phenotypic change associated with the HA protein of the third-wave viruses compared to the first-wave isolate. A comparison of the viruses' relative abilities to hemagglutinate chicken, turkey, or guinea pig red blood cells indicated a relative increase in affinity to α-2,6-linked sialic acids and a concomitant decrease in the ability to bind to α-2,3-linked sialic acids for the majority of the third-wave isolates. This alone may have increased the ability of the virus to infect the human upper respiratory tract, accounting for the greater replication of A/687 in HAE cells. That the lost affinity for the α-2,3-sialic acids is observed in both second- and third-wave isolates would implicate one or more of the previously reported variations in the HA I32L, D97N, S185T, E374K, and S451N (27). A recent publication by de Vries et al. (50) indicated a change in receptor binding caused by an S185T mutation. We also note the recent publication from Cotter et al. (51) showing that later isolates of A(H1N1)pdm09 virus contained pH-stabilizing mutations in HA which enhanced their replication in the ferret upper respiratory tract. This mutation at HA residue E374K (H1 numbering) is present in 5 of 6 third-wave viruses in our subset (Table 1) and may also have contributed to the increased replication in HAE cultures we observed (Fig. 2b).

There may also be a contribution of NA to the increased propagation of third-wave virus in HAE (Fig. 5c). All of the second- and third-wave viruses in our cohort had NA mutations V106I and N248D compared to first-wave A/195. These are proposed to enhance viral stability, through modifications in pH tolerance under acidic conditions, although we did not assess this phenotype (52). However, we did observe an increase in the ability of virus with third-wave NA to retain its infectivity in human mucus, a property linked with efficient replication in HAE cells and transmission in ferrets (23). Several other research groups have already investigated variations between individual isolates of the A(H1N1)pdm09 lineage. These publications described a few selected isolates from mild, severe, or fatal cases and characterized their phenotypes in various animal models without considering their broader phylogenetic relationships (41, 53–55). A correlation has sometimes been observed between the outcome of infection in animal models and the severity of the human case from which the isolate was obtained; this was not the case in our study. Rather, we found increased replication in primary human cells was often accompanied by decreased virulence in the murine model. It is well recognized that the mouse is not a good model host for human-adapted influenza viruses.

Our work suggests that changes to internal viral proteins, including NS1 and PA, occurred during evolution of the third-wave viruses and adapted them for increased replication in human cells. Others have also recently suggested that mutations have occurred in the virus polymerase, since its transfer to humans may enhance replication or transmission (56, 57). These previously described mutations may contribute to some of the enhanced polymerase activity we measured for the A/687 third-wave virus polymerase, but our *in vitro* polymerase reporter system also indicated that the N321K amino acid change in the PA protein, not previously reported, drove enhancement of viral polymerase activity. How it may achieve this is unclear. PA is known to interact with host factors, such as transcriptional modulator of RNA polymerase II (RNAPII) hCLE (58, 59) and the minichromosome maintenance complex (60). The PA gene segment of A(H1N1)pdm09 virus was originally derived from an avian source during the formation of the TRIG internal gene cassette. Bussey et al. already described three amino acids (85I, 186S, and 336M) present in the A(H1N1)pdm09 PA that are not usually present in PA of avian viruses. They concluded that these three mutations together may have contributed to the ability of this polymerase complex to function better in mammalian cells. The mutation at residue 336 is situated close to amino acid 321 on the crystal structure (61).

PA 321K was, until now, rare in swine-adapted influenza viruses, occurring in only 1.85% of sequences of all influenza virus A subtypes and in 3% of swine H1N1 viruses before the emergence of A(H1N1)pdm09 in 2009. It will be interesting to see if there is an increase in the occurrence of a lysine at this position in swine as the A(H1N1)pdm09 virus circulates in pigs or is reintroduced to this host through contact with humans after the third wave. Only 2.06% of avian isolates have PA 321K. This amino acid does not occur in the PA segment of seasonal human influenza viruses, but the prevalence of the lysine variation in human isolates of pandemic H1N1 increased sharply as sequences from later in the pandemic were submitted to NCBI, supporting its prevalence during and after the third wave. We expected the advantage conferred by this single PA mutation engineered alone to be subtle, and therefore we analyzed the effect by using a competition assay rather than by direct comparison of growth curves. Previously, we showed that the NA mutation H275Y in first-wave virus conferred a replicative cost that was not detected by growth curve analysis but only by competition assay (62). Indeed, we found that virus with the single PA change 321K outgrew 321N virus in 3 biological replicates of HAE cultures. This type of fitness assay could be useful in predicting the selection of mutations that confer subtle advantage.

The other gene segment we investigated in detail here was that which encodes the NS1 protein. Interestingly, we found a cluster of mutations in NS1 that slightly enhanced its ability to control the innate immune response, including IFN and other cytokines. In the murine model, decreased virus titer in the lungs early in infection with third-wave viruses may also have contributed in part to decreased IFN production. However, in human airway cultures where third-wave virus replicated robustly, we observed decreased IP-10 production in comparison with A/195. We attribute this to changes in NS1, because other known antagonists of the interferon response, such as PB1-F2, were not different between these two viruses. Moreover, a 6:2 recombinant virus with HA and NA from the third wave on a first-wave internal gene constellation induced higher IP-10 and other cytokine responses in HAE cells than whole first-wave virus (data not shown). This suggests that the differences in cytokine response were not accounted for in this case by changes in receptor binding specificity that affected ciliated versus nonciliated cell tropism in the HAE cultures, as suggested by Ramos et al. in 2011 (63). One explanation for our findings is that the NS1 protein had acquired some ability to bind to host cell factor CPSF30 and inhibit host mRNA processing, a mechanism by which other human-adapted influenza viruses have enhanced their control of the human innate immune response (64, 65). Indeed, exogenous expression of the A/687 NS1 gene inhibited the expression of a reporter gene from a polymerase II promoter significantly more efficiently than the NS1 gene from A/195 did (data not shown). This property is associated with an ability to bind and inhibit CPSF30 and may account for the difference we observed in heterologous control of induced interferon (Fig. 4c). Hale et al. already predicted some mutations in A/California/04/2009 virus by which this could be achieved, one of which is common to the A/687 NS1, G189D (7). That study also reported rather subtle effects on virus replication for viruses that were engineered to have CPSF30 binding. Notably, one virus isolated in the third wave, A/689, which phylogenetically lies closer to second-wave viruses, was associated with higher cytokine levels than other third-wave viruses in mouse lungs.

In conclusion, using a collection of A(H1N1)pdm09 viruses chosen based on phylogenetic divergence, we have shown that amino acid changes in HA, NA, NS1, and PA in later waves led to functional changes in individual viral genes that conferred increased replication in primary human airway cells, suggestive of human adaptation. The increase in viral fitness overall may facilitate increased transmission, as suggested by Doriggati and Ferguson, but the lack of viral genetic differences between severe and community influenza cases suggests these changes are not on their own sufficient to confer severe disease (16), especially as there is evidence of high levels of asymptomatic or unreported illness during the first three waves of A(H1N1)pdm09 virus activity in the United Kingdom (12). Infection of those people predisposed to more severe disease may have been more likely in the third wave because of the differences in viral replication, host immune modulation, and viral persistence in the host and the external environment (66–68).
